# Fractional quantum Hall effects in In_0.75_Ga_0.25_As bilayer electron systems observed as “Finger print”

**DOI:** 10.1038/s41598-019-43290-8

**Published:** 2019-05-15

**Authors:** Syoji Yamada, Akira Fujimoto, Siro Hidaka, Masashi Akabori, Yasutaka Imanaka, Kanji Takehana

**Affiliations:** 1 Osaka Institute of Technology, 5-16-1, Omiya, Asahi-ku Osaka 535-8585 Japan; 2 LT Center, Osaka University, 1-1, Machikaneyama, Toyonaka, Osaka 560-0043 Japan; 3 Japan Advanced Institute of Science and Technology, 1-1, Asahidai, Nomi, Ishikawa 923-1292 Japan; 4 National Institute for Materials Science, 3-13, Sakura, Tsukuba, Ibaraki 305-0003 Japan

**Keywords:** Two-dimensional materials, Quantum Hall

## Abstract

Observations of fractional quantum Hall (FQH) plateaus are reported in bilayer electron gas system in wide (>80 nm) In_0.75_Ga_0.25_As wells. Several *q*/*p* (*p* = 5, 3, and 2, *q* > 5) QH states are confirmed at high temperatures (~1.6 K) when the critical conditions including an electron density imbalance as well as a dynamical resistance behavior at the bilayer-monolayer transition are properly satisfied. The former leads to a quantum limit in either of the layers and the latter might bring a meta-stable nature into FQH phenomena. Such a behavior occurs as a probability process associating with impurities or defects in the wells, they inevitably reflect the local structural landscapes of each sample. This is verified by the new finding that the kinds of fractional plateaus (what set of fractional filling factors) appeared are different depending on the samples, that is, they are the “finger print” in each sample.

## Introduction

Integer^1–3^ and fractional^4,5^ quantum Hall effects (IQHEs and FQHEs) are remarkable phenomena arising from the distinctive dynamics of two-dimensional electron gas (2DEG) under strong perpendicular magnetic fields. IQHE is related to energy gaps in the single-particle density of states comparable to the cyclotron energy, *ℏω*_*c*_. In FQHE, however, the gaps in the excitation spectrum, which are a direct result of the intra-layer Coulomb interaction, play an important role. This effect is a so-called incompressible phase of quantum liquid state described by Laughlin wave function^6^. In a standard single 2DEG which is confined to a low-disorder (high-quality) GaAs well and occupies a single subbnad, the FQHE is mostly observed at the conditions of lower Landau-level (LL) filling factors *ν* < 2, where the Fermi energy (*E*_*F*_) locates within the lowest index (*N* = *0*) spin-split LLs. The strongest ones are generally seen at the *q*/*3* fractional filling levels, such as *ν* = *1*/*3*, *2*/*3*, *4*/*3* and *5*/*3*. When *E*_*F*_ lies in the second LLs (*N* = *1*, *2* < *ν* < *4*), however, the equivalent *q*/*3* states (*ν* = *7*/*3*, *8*/*3*, *10*/*3* and *11*/*3*) are becoming much weaker^7,8^. In even higher LLs (*4* < *ν*) such as *ν* = *13*/*3*, *14*/*3*, *16*/*3* and *17*/*3*, the FQH states are essentially not observed^9–11^.

Fabrication of multiple 2DEG layers in close proximity allows the controlled introduction of additional degree of freedom associated with the third dimension. The double quantum well (DQW) or the wide-single quantum well (WSQW) is the simplest of these structures and preserves both high electron mobility and external gating of the electron density in each layer. In fact, several novel findings have been reported in the 2DEG bilayer transport. First, for example, interlayer Coulomb interactions in a multilayer structure have been predicted to lead even denominator FQH states. This prediction has been followed by the successive observations of new FQH states at *ν* = *1*/*2* for 2DEG systems in a WSQW^12,13^ or a DQW^14^. Second example nearly concerned with this article is the report^15,16^ on the stability discussion of the *q*/*3* states in GaAs WSQW systems: The demonstrated *R*_*xx*_ data show that the *q*/*3* states are confirmed to be stable even at filing factors up to *ν* = *17*/*3*, when *E*_*F*_ lies in a ground state (*N* = *0*) LLs regardless of whether the ground LL belongs to the upper or lower (symmetric or anti-symmetric) 2DEG subband. Instead, the FQH states with even-denominator fillings of *ν* = *5*/*2* and *7*/*2* observed often in a narrow (30 nm) quantum well are found to be absent. So that, in this sense, the bilayer 2DEG system might be more suitable than the monolayer to observe FQH states.

As is well known, especially FQHE phenomena have been studied mostly in high quality GaAs 2DEG system, in which the disordered potential fluctuation, 〈(*ΔV*)^*2*^〉^*1*/*2*^ ~ *ħ*/*τ*_0_ ~ *ħe*/*m* * *μ*, could be suppressed to very much smaller value than the energy gap between the Laughlin states. Mainly owing to the recent progress in high quality crystal growth technologies, FQHE states have recently been reported in a variety of new semiconductor materials other than high-quality GaAs: For example, various FQH plateaus have been reported, for example, in strained Si quantum wells (ν = *2*/*3* and *4*/*3*)^17^, CdTe (*4*/*3*, *5*/*3*, *7*/*3*, *8*/*3*)^18^, Mg_x_Zn_1−x_O/ZnO hetero-structures (*1*/*3* and*1*/*2*)^19,20^, Si/SiGe field-effect-transistor (*q*/*3*, *q*/*5*, *q*/*7* etc.)^21^, CdMnTe (*4*/*3*, *5*/*3*, *7*/*5*, *8*/*5*)^22^, and Ge quantum wells^23^. Note that the materials described above are almost the compound semiconductors and alloy materials are very rare.

As is imagined, there have been not so much works on QHE phenomena in In_x_Ga_1−x_As alloy hetero-junction system. Most reports have limited their interests to fundamental IQHE behaviors^24,25^ and/or to scaling problems^26–31^ in that regime and hence there have almost no studies on FQH phenomena in In_x_Ga_1−x_As 2DEGs. The main reason is an alloy scattering which becomes maximum especially at Indium content of x ~ 0.5 (lattice-matched to InP substrate) and hence results in high-disorder and low average mobility in those 2DEGs. Another origin we consider is an in-plane structure inhomogeneity due to anisotropic lattice relaxation. This often appears not as an electron density inhomogeneity but as an in-plane mobility anisotropy in In_x_Ga_1−x_As hetero-junctions. Probably due to these reasons, the disorder in the In_0.5_Ga_0.5_As 2DEGs still remains at relatively higher level even at low temperatures. That is, the average mobility when x ~ 0.5 typically reaches as high as ~5 m^2^/V · sec. and likely saturates at relatively high temperatures as 5–10 K.

The In_x_Ga_1−x_As 2DEG wafers adopted in this paper have a nominal Indium-content of 0.75 and hence have a larger low-temperature mobility up to ~40 m^2^/V · sec^32^ in the monolayer 2DEG samples. But, we are here measuring the bilayer samples with a variety of layer spacing and modulation-doping conditions, and their typical mobility is roughly 10–15 m^2^/V · sec. As described later, in such bilayer systems, there found a certain mechanism related to the even stable appearance of *q*/*3* FQHE plateaus^16^ and/or resistance instability with spike noise and hysteretic behaviors, which might help the occasional observation of the FQHE states. We indeed, for the first time in this alloy system, report here the observations of several FQHE plateau-like features (plateaus/shoulders) in the 2DEG bilayer system in In_0.75_Ga_0.25_As/In_0.75_Al_0.25_As heterojunction. In addition, we confirmed sample-dependent appearance of the FQHE behaviors. This is reasonably expected from the instabilities associated with the bilayer-monolayer transition, since the instability process generally depends strongly on the sample. We thus attribute the main origin of this new observation first to the grossly high-mobility realized in our bilayer 2DEGs, which means a relatively low disorder. Second, the *imbalanced* electron density distribution crucial for giving the quantum limit condition at one interface as well as the metastable nature frequently seen at bilayer-monolayer transition are considered to facilitate the observation.

## Sample Preparation

**Figure 1:**
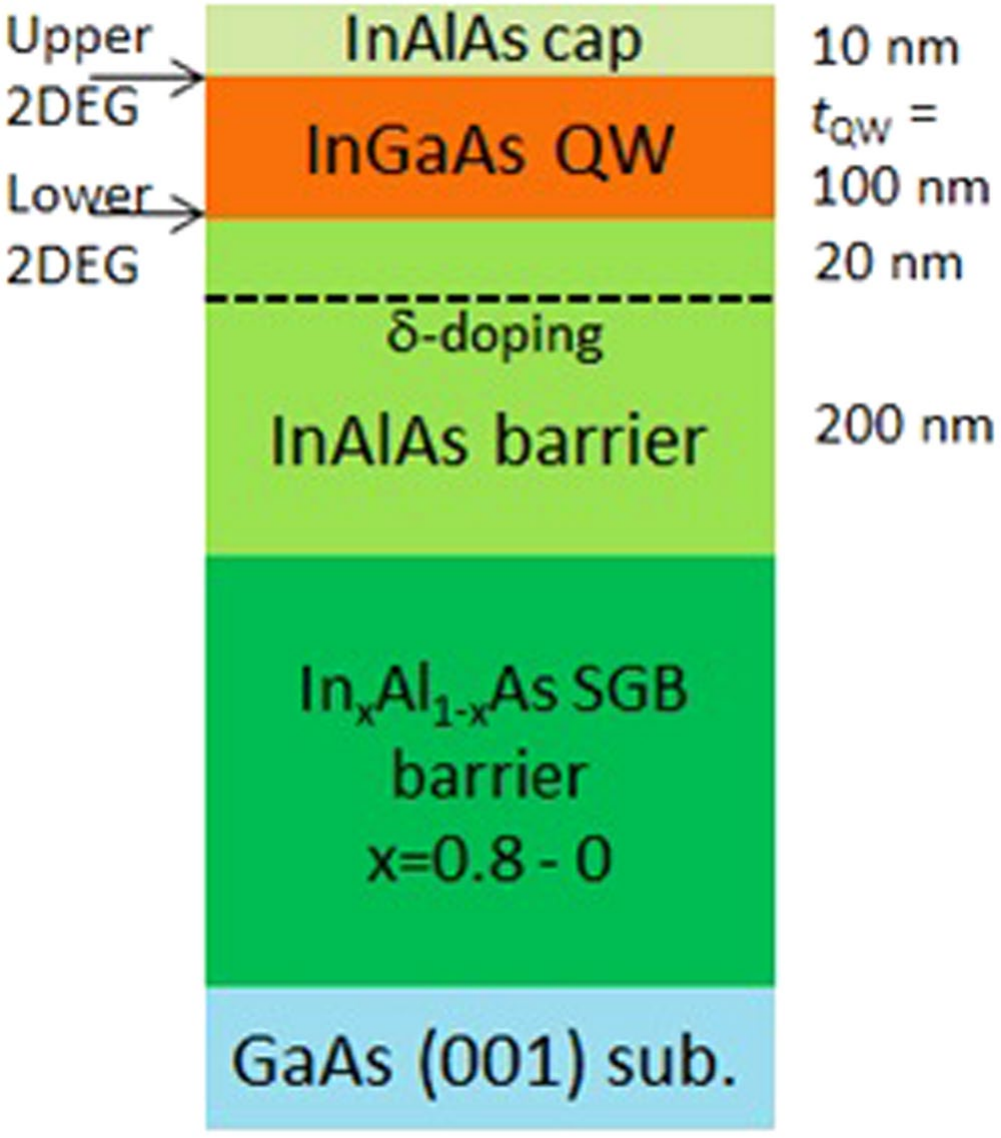
Schematic layered structure of samples A–C. The distance between the 2DEG profile peaks is ~90 nm or less.

We have measured and compared four In_0.75_Ga_0.25_As well Hall bar samples. The Hall bar has a length and a width of 600 and 50 μm, respectively, with current and voltage probes, and the distance between the voltage probes is 200 μm. They are fabricated by a standard photolithography and dilute sulfuric acid etching technique. Three of them (samples A, B and C) are fabricated from one wafer, which has a wide (*t*_*QW*_ = 100 nm) well (channel) on the top surface of a non-doped In_0.75_Al_0.25_As barrier with a thin (10 nm) In_0.75_Al_0.25_As cap. The schematic layer structure is shown in Fig. 1. The fourth sample (sample D) has a buried In_0.75_Ga_0.25_As well located at 80 nm below the surface and the well width is *t*_*QW*_ = 40 nm. Here we adopted In_x_Al_1−x_As step-graded-buffer (SGB) to expand the lattice constant by utilizing metamorphic molecular beam epitaxy (MBE) growth^32^. Although the In_0.75_Al_0.25_As/In_0.75_Ga_0.25_As material system is not so widely studied, the detailed growth recipes are made open for several decades ago by different two groups including ours^30–32^. In a final processing step, we have covered the entire surface of the bar channel (600 × 50 μm^2^) by a Ti/Au top-gate (~20 nm thick) evaporated via ~30 nm thick Al_2_O_3_ thin film attached by atomic-layer-deposition (ALD) on the In_0.75_Ga_0.25_As channel after removing the cap. In this sense, there are no electron density inhomogeneity^33^ except the microscopic one due to the alloying nature of In_0.75_Ga_0.25_As, which is averaged in macroscopic magnetoresistance measurement and hence gives no influence to QHE phenomena.

The potential distribution having two opposite triangle forms at both the interface (similar to the GaAs WSQW samples^12,13^) is expected in all the samples: That is, the realization of 2DEG bilayer is indeed confirmed in the same magnetoresistance (MR) method as already reported in our initial bilayer samples^34^. The areal electron densities are estimated from the low-field MR signals. “Low field” means in this material system is roughly ≤5 Tesla, where *R*_*xx*_ signal can be regarded as a sinusoidal function of inverse magnetic field, *1*/*B*. At V_g,top_ = 0, *n*_*s*,*lower*_/*n*_*s*,*upper*_ ~ 2 (*n*_*s*,*lower*_ ~ 4.2 and *n*_*s*,*upper*_ ~ 2.3 × 10^11^ cm^−2^) for the three surface well samples (A–C) and ~4 (~3.5 and ~0.8 × 10^11^ cm^−2^) for the buried well one (D) were confirmed. Here *n*_*s*,*upper*_ and *n*_*s*,*lower*_ are the densities of upper and lower 2DEGs, respectively. By using the top-gate in the samples, we can deplete the upper 2DEG by applying a deep negative gate voltage, V_g,top_ < 0. In other words, we can realize the bilayer-monolayer transition by tuning the V_g,top_. However, we cannot vary *n*_*s*_ (V_g,top_) and *ν* (magnetic field *B*) independently, since unfortunately the fabrication of back-gate was not succeeded in this type of samples.

## Results

### Surface 100 nm well samples (samples A, B, and C)

#### Magneto-resistances, *R*_*xx*_, *R*_*xy*_ and areal densities n_s_s depending on V_g,top_

**Figure 2:**
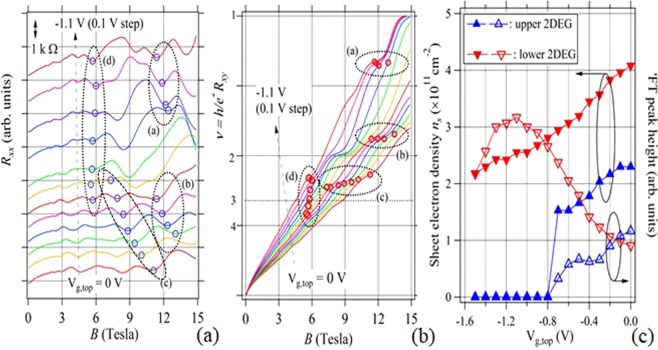
(**a**) *R*_*xx*_s (shifted vertically with each other for clarity), (**b**) *R*_*xy*_s as functions of *B* with a parameter V_g,top_, and (**c**) *n*_*s*_s and FT peak heights as functions of V_g.top_ for the 2DEG bilayers in sample A. V_g,top_ was changed from 0 to −1.1 V. In (**a**,**b**), four data point groups of (**a**–**d**) were indicated by dotted ellipsoids, which are utilized to make dip-plateau/shoulder plots in Fig. 3(a–d) varying with V_g,top_ and hence depending on *B*.

Figure 2(a,b) show MR curves, *R*_*xx*_ and *R*_*xy*_, respectively, obtained by changing V_g,top_ (=0~−1.1 V) in sample A. Here the upper 2DEG appears at the In_0.75_Ga_0.25_As well top surface inversion layer, whereas the lower 2DEG exists at the lower interface of the well. We have deduced *n*_*s*,*upper*_, *n*_*s*.*lower*_ and associating Fourier transform (FT) peak heights from the fast FT analysis of the low-field part of *R*_*xx*_ in (a),and plotted as functions of V_g,top_ in Fig. 2(c). Note here that at V_g,top_ ~ −0.7 V, *n*_*s*,*upper*_ suddenly falls to zero, that is, a bilayer-monolayer transition occurs at the V_g,top_. Moreover, at V_g,top_ < −0.7 V, the FT peak of the lower 2DEG takes a maximum suggesting that interlayer scattering between the 2DEGs becomes highest just after the transition. Since this is related to the charge transfer between the layers, we later discuss the behaviors of *n*_*s*_s and FT peak heights in detail. Although *R*_*xy*_s in Fig. 2(b) might show complicated behaviors, we can see some clear integer plateau-like features at *ν* = 1 (V_g,top_ = −0.8, −0.9, −1.0, −1.1 V at ~15 T) and 2 (−0.8 and −0.9 V at ~8 T), respectively. Besides the integer plateaus, some irregular plateaus/shoulders as indicated by four ellipsoids, (a–d), could be observed at non-integer *ν*, which seem not simply the static transient processes between the integer *ν* s associated with V_g.top_ variation.

#### Dip (minimum) – plateau/shoulder plot

**Figure 3:**
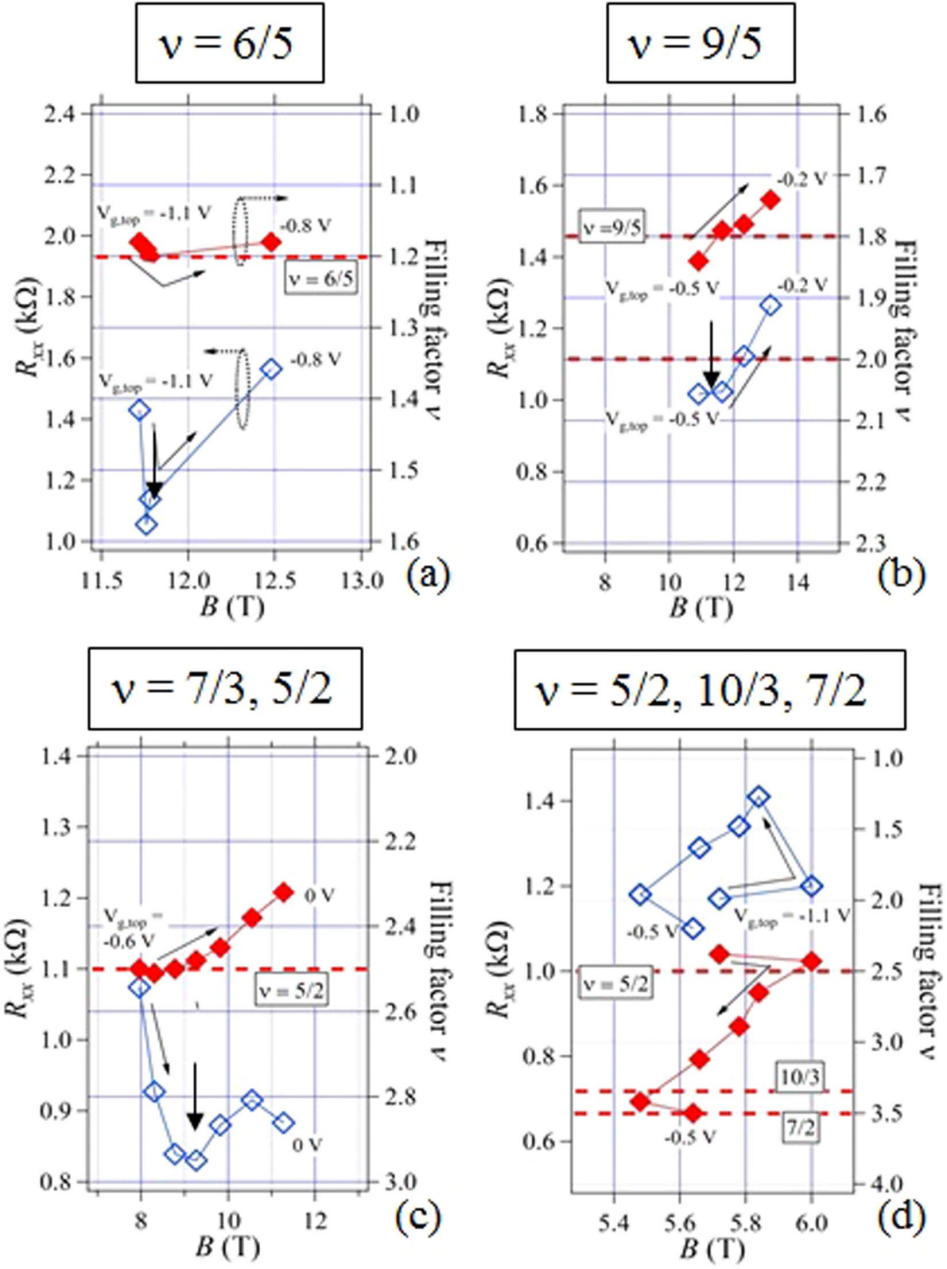
*R*_*xx*_ dip – *R*_*xy*_ plateau/shoulder pairs as functions of magnetic field (*B*) observed at various filling factors, (**a**) *ν* ~ 6/5, (**b**) ~9/5, (**c**) ~7/3 or ~5/2, (**d**) ~5/2 and 10/3 or ~7/2 in sample A. The panels (a–d) are created from the data sets in the ellipsoids, (**a**–**d**) in Fig. 2(a,b). Note here the *ν* values approach to certain fractional ones and also *R*_*xx*_s decrease or show minima (arrows) at the same *B* regions, suggesting metastable states.

In order to inquire the details of such *R*_*xx*_ and *R*_*xy*_ behaviors, we have made a tracking of *R*_*xy*_ plateaus/shoulders with corresponding *R*_*xx*_ dips (minimums) for the non-integer *ν* dip-plateau/shoulder pair groups, (a~d), shown in Fig. 2(a and b). The dip-plateau/shoulder points divided into the four groups are indicated by open circles in the dotted ellipsoids in Fig. 2(a and b). From those data, we have deduced *R*_*xx*_ values as well as corresponding *R*_*xy*_ (*ν*) values from Fig. 2(a and b), respectively and the plots in Fig. 3(a–d) were created to show the results of tracking. They can thus be regarded as an expanded presentation of the data in the ellipsoids in Fig. 2(a and b). They show some irregular behaviors such that *ν* (solid diamond) approaches some specific fractional values and corresponding *R*_*xx*_ (open diamond) decreases or takes minima at the almost same field, as indicated by arrows. That is, metastable fractional plateau-like features could be seen related to the values of *ν* = *h*/*e*^*2*^*R*_*xy*_ = 6/5, 9/5, 5/2–7/3 and 5/2–10/3 in Fig. 3(a–d), respectively. The multi-value behavior recorded in Fig. 3(d) can also be considered as a right proof of hysteretic nature probably related to the meta-stability. It is very much interesting that even-denominator shoulders are also observed as well as odd-denominator ones. Even denominator plateau/shoulder has usually been observed so far in some specific GaAs bilayer samples^12–16^. We will discuss the details later.

We then investigate the other two samples, B and C (from same wafer) and make a comparison with those of the first sample A. In sample B, the plateau of *ν* = 6/5 is missing, but other three fractional plateaus are observed in a similar manner. Especially, *ν* = 7/3 new plateau seems to appear. For sample C, we have confirmed the same four fractional plateaus as found in sample A and their appearance manners are much resemble to the cases in sample A. Those fractional shoulder/plateau data are found in Supplementary Fig. S1. Besides those fractional plateaus, we confirmed further 7/5 plateau in sample C, and 16/3 and 11/2 in sample B and C (See Supplementary Fig. S2). In all the additional *ν* shoulders/plateaus, we have confirmed the decrease or the minima in *R*_*xx*_.

To summary, we have totally nine (6/5, 7/5, 5/3, 9/5, 7/3, 5/2, 7/2, 16/3 and 11/2) different fractional shoulders/plateaus in the above three samples and they are listed in Table 1. Related to this result, we can conclude that those fractional phenomena are observed not accidentally but reproducibly. The phenomena themselves are thus not fragile but robust. However, what kind of plateaus/shoulders appear depends on the sample, since the appearance seems to be related to the metastable process, where the carrier movement occurs via impurity or defect levels etc. unique to each sample. In this sense, they seem to appear as “finger print” of each sample.

**Table 1:** Summary of fractional QH plateaus/shoulders observed in three different Hall-bar samples fabricated from the same wafer.

Filling factor	1 < *ν* < 2				2 < *ν* < 3		3 < *ν* < 4	5 < *ν* < 6	
Sample	6/5	7/5	5/3	9/5	7/3	5/2	10/3 or 7/2	16/3	11/2
A	√			√		√√	√		
B			√	√	√	√	√	√	√
C	√	√		√		√√	√	√	√

Observed fractional plateaus/shoulders are different depending on the sample.

### Buried 40 nm well sample (sample D)

**Figure 4:**
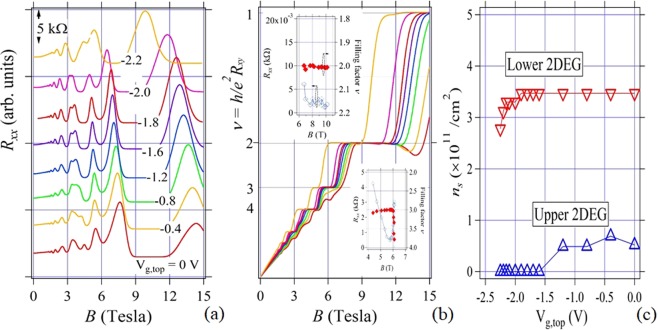
(**a**) *R*_*xx*_s (shifted for clarity), (**b**) *R*_*xy*_s as functions of *B* with a parameter V_g,top_, and (**c**) 2DEG areal densities n_s_ vs V_g,top_ in sample D. Insets in (**b**) are *R*_*xx*_ dip – *R*_*xy*_ shoulder (plateau) plots almost at the integer filling factors *ν* ~ 2 and 3 as functions of *B* obtained from the data in (**a**,**b**). In the figure (**b**), there are no fractional plateau-like structures between the integer ones in contrast to the results in Fig. 2(b).

In contrast to the results in the samples A - C, we have obtained no fractional behaviors in the MRs in the buried 40 nm well sample (sample D). We show *R*_*xx*_, *R*_*xy*_ and n_s_ dependencies on V_g,top_ in Fig. 4(a–c) when V_g,top_ is changed. Insets in Fig. 4(b) are the *R*_*xx*_ dip - *R*_*xy*_ plateau/shoulder plots for the filling factors, *ν* = 2 and 3 similar to those in Fig. 3. Initial n_s_ balance is different (~0.8 and ~3.5 × 10^11^ cm^−2^ for upper and lower 2DEGs) from that in samples A-C and the bilayer-monolayer transition occurs at V_g,top_ ~ −1.2 V in this sample. We have searched fractional dips in *R*_*xx*_ and corresponding plateau/shoulders in *R*_*xy*_ in entire V_g,top_ regions, but there found no such phenomena in between the integer features in sample D. As seen in the insets in Fig. 4(b), it is found that for all the cases of *R*_*xy*_ values near *ν* ~ 2, 3 and 4, *R*_*xx*_s take the proper minima at the same *B* region. This result is typical IQHE phenomenon and suggests the well thickness-related origin for the FQH states in the samples, A–C. It is thus supposed that strong Coulomb interaction between the 2DEGs in narrow well sample might disturb the temporal formation of the FQH states. Actually, although another narrow well (20 nm) buried sample show no fractional behaviors, fractional plateau-like ones are confirmed also in 80 nm well sample.. Most different structure parameter among the samples treated here is thus the well thickness *t*_QW_, which might determine the strength of the Coulomb interaction between the 2DEG layers directly.

## Discussion

### Conditions to observe FQH behaviors in our heterojunction 2DEG

In general, as is well known widely, fractional QHE experiments have been done at low temperatures less than ~0.3 K by using high quality (low areal electron density with very high mobility at such temperatures as above) GaAs/AlGaAs 2DEGs. This is necessary to observe a very small energy gap between ground and excited Laughlin states^6^. The energy is represented by *Δ* = *E*_*g*_/*2* ~ (*Ce*^*2*^/*2κ* *l*_*B*_) or ~(*Ce*^*2*^/*2κ*)(*n*_*s*_)^*1*/*2*^, where *κ* is a permittivity, *l*_*B*_ = (*ℏ*/*eB*)^*1*/*2*^ a magnetic length, *n*_*s*_ an areal density and *C* is usually a constant less than 0.1. The value is defined experimentally by measuring temperature dependence of *ρ*_*xx*_ (T) ∝ *exp* (−*Δ*/*k*_*B*_*T*) at plateau region. For example, *C* becomes ~0.003–0.03, if we investigate the values obtained in the various experiments^35–41^. If we assume the minimum value, *C* ~ 0.003, *κ* ~ *10ε*_*0*_, (*ε*_*0*_: vacuum permittivity) and *l*_*B*_ ~ *10* nm (*B* ~ 9 Tesla) or *n*_*s*_ ~ 5 × 10^15^ m^−2^, then *Δ* becomes 0.27 meV (~3.1 K) or 0.19 meV (~2.3 K). Even for the minimum experimental *C*, *Δ* is still larger than the temperature (1.6 K) which we adopted in our high-field measurements.

Another important condition is that *Δ* should be larger than the disordered potential fluctuation in the samples, that is, *Δ* = *E*_*g*_/*2* ≥ 〈(*ΔV*)^*2*^〉^*1*/*2*^. This fluctuation is estimated by the equation *ħ*/*τ*_*0*_ ~ *ħe*/*m* * *μ*, which gives 0.3 meV ~ 3.6 K if we assume m* = 0.04 *m*_*0*_ and *μ* = 10 m^2^/V · sec. This value is larger than *Δ* ~ 2.3 K estimated above and hence the observation of FQHE seems pessimistic. However, since *C* might be larger than the minimum value in our materials, the orders of the two parameters becomes almost the same and thus we might have some opportunities to detect the FQH-related phenomena. In addition, if we can utilize some meta-stable conditions or instabilities unique to and sometimes appearing in bilayer 2DEG systems as discussed below, the possibility to observe FQH-related phenomena becomes even larger. Note also here that the mobility of the GaAs bilayer samples in which the *q*/*2* plateaus are sometimes observed is not so high and is less than 50 m^2^/V · sec^14^.

### Instabilities between virtual levels in *R*_*xy*_ by dynamic charge transfer at the layer transition leading “finger prints” nature

We pay attention here again to the details of the abrupt depletion of *n*_*s*,*upper*_ in Fig. 2(c). It is found that when we close from zero gate-bias (V_g,top_ = 0) to the transition region at V_g,top_ ≈ −0.7 V, *n*_*s*,*upper*_ shows a little shoulder and drops abruptly to zero at V_g,top_ ~ −0.8 V. Simultaneously *n*_*s*,*lower*_ slightly decreases than linearly, then makes a small hill just after the depletion and decreases gradually. Corresponding FT Peak heights for upper and lower 2DEG oscillations (open symbols, right axis) are also plotted against V_g,top_. We then notice that the upper 2DEG peak height also makes a shoulder at the *n*_*s*,*upper*_ shoulder just before the depletion. The lower 2DEG peak height takes a maximum with the *n*_*s*,*lower*_ small hill just after the depletion. Since the FT peak itself represents the relative measure of the scattering strength of the 2DEG layer, we might regard the variation of FT peak as the amplified signal of *n*_*s*_ dynamical behavior. Such complex behaviors of *n*_*s*_s and SdH oscillations peak heights are suggesting the occurrence of the delicate interlayer charge transfer process between the 2DEG layers. The FT peak height increase is considered to be the increase of the interlayer scattering due to the electron transfer between the layers. That is, in our case, the dynamic charge transfer likely occurs initially from the lower 2DEG to upper 2DEG just before the transition and then it occurs inversely from the upper to lower just after the transition, although the reason is not clear at present. Similar type (but more simple) charge transfer accompanying the bilayer-monolayer transition has already been discussed widely in GaAs bilayer systems. In the experiments reported in refs ^42,43^, *n*_*s*,*lower*_ increases with decreasing V_g,top_ just before the *n*_*s*,*upper*_ depletion associating with a faster *n*_*s*,*upper*_ decrease than linearly. The charge transfer in this case seems uni-directional. Although the fine features are not equal between the GaAs and our cases, there could be some charge transfer phenomena occurring between the layers at the vicinity of the bilayer-monolayer transition.

It should thus be noted here that this type of transfer may lead to an instability, in which *R*_*xx*_ (and hence *R*_*xy*_) often reveals hysteretic behavior against field sweep direction^44,45^, for example. The origin has been believed to be a charge movement from/into a parallel low-mobility 3D path^46^ or a parallel 2DEG layer^47^. If we consider that such instabilities often occur even at low temperatures, the activation energies of which are small enough and could be comparable with that (*Δ* ~ 3.1 K) of FQHE excited states. In such a case, fractional QHE phenomena could occur easily with the help of the virtual levels corresponding to the resistance (*R*_*xx*_ and *R*_*xy*_) difference in between the bilayer and monolayer states. They thus inevitably reflect the scattering processes in both the *R*_*xx*_ and *R*_*xy*_ cases. The scattering process is actually related to the microscopic scattering paths determined from the arrangement of impurity, defect and alloy disorder. The arrangement itself is of course highly sample dependent and thus the details of the sample, that is, the detailed but dynamic landscapes of the impurities etc. affect the FQHE phenomena. In this sense, FQHE phenomena (*R*_*xy*_ plateaus) especially in our case could become a supersensitive sensor for the impurity/defect landscapes and thus show the “finger print” nature of each sample. Actually, the well-known “finger print” in magneto-conductance has been observed in narrow wires with smaller scales than phase coherence length (*l*_*ϕ*_) of the sample electrons^48,49^. In our case, however, not the sample scale (large enough than *l*_*ϕ*_) but the small energy scale of FQHE states themselves could give the high sensitivity for the energy difference between the scattering paths. The fact that the *q*/*3* states have become more robust at the layer transition region in GaAs bilayers discussed below again might be interpreted by similar origins

### Quantum limit condition (Comparison with GaAs 2DEG bilayer systems)

As is well known in the past few decades, FQH plateaus with odd and even denominator have been confirmed widely in various GaAs samples including the bilayer ones. However, we focus on the work in which the stability of the *q*/*3* FQH states has been discussed^16^. It is found that the *q*/*3* states are stable and strong even at high fillings, as long as the Fermi level *E*_*F*_ lies in a ground state (Landau number, *N* = 0) Landau level of either of the two electric subbands of the bilayer, regardless of whether that the Landau level belongs to the symmetric or the antisymmetric (lower and upper) subbands.

**Figure 5:**
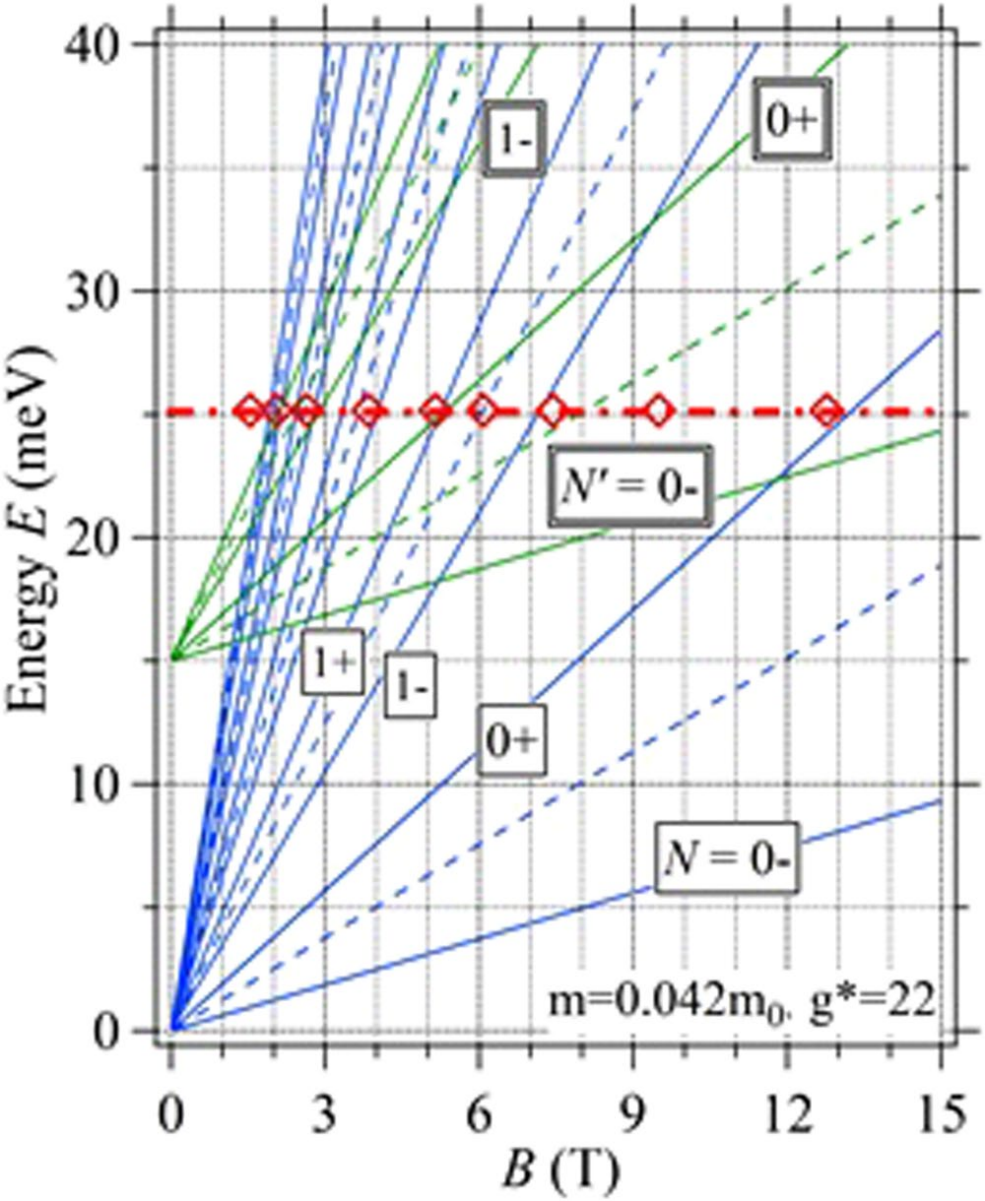
Fan diagram in sample A when V_g,top_ = 0 calculated assuming a simple model (non-parabolicity and a polaron effect are ignored). *N*s are the landau numbers and the open diamonds shows the *R*_*xx*_ peak position obtained in the experiments when V_g,top_ = 0.

In our 2DEG bilayer samples, we can realize the bilayer-monolayer transition by decreasing V_g,top_. Just before the transition, we can expect to occur the quantum limit condition (*E*_*F*_ ~ *E*_*0*_ (*N* ~ 0 Landau level)) in the upper 2DEG subband. In order to confirm this situation, we have calculated a simple Landau fan diagram in sample A and shown in Fig. 5. Fan curves are written by assuming the electron effective mass at the Fermi level and the effective g-factor to be m*/*m*_*0*_ = 0.042 and g* = 22, respectively in the equation, *E*_*N±*_ = *ħω*_*c*_(*N* ± *δ*) for integer *N*, where *δ* = (*1* − g*m*/*2m*_*0*_)/*2*. Open diamonds are the plot of the field values of *R*_*xx*_ peak at V_g,top_ = 0 obtained in the experiment. Although the non-parabolicity and the polaron effect are neglected, several data points can be fitted by the integer Landau level curves. As seen in this figure, the quantum limit condition that *E*_*F*_ lies between *N*’ = 0+ and 0− lines of the upper 2DEG is realized in the wide field region until the upper subband electron is depleted just before the bilayer-monolayer transition (at *E*_*F*_ ~ 15 meV). In this sense, the *E*_*F*_ configuration very much resembles that discussed in ref.^16^ is indeed seen also in our samples.

### Coulomb interactions and well widths

**Figure 6:**
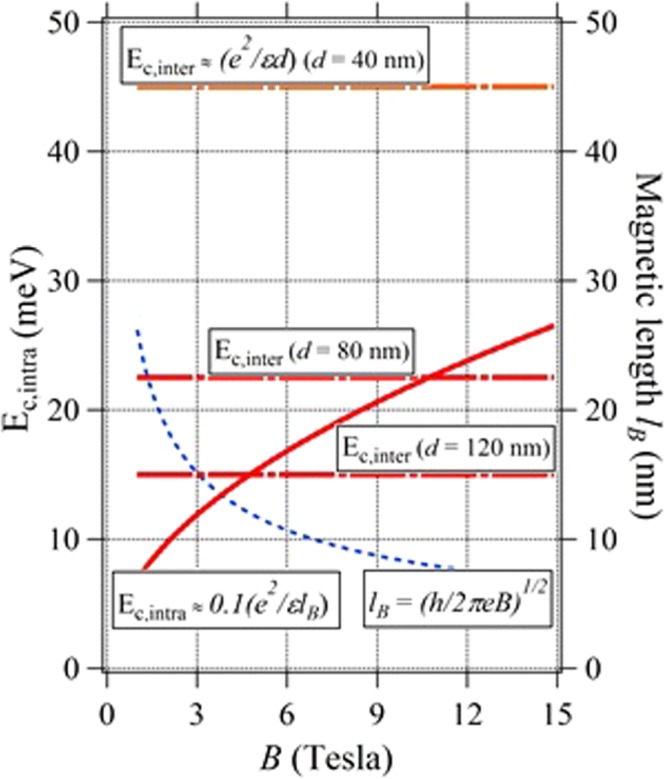
Energy and length parameters relevant to Coulomb interactions in our samples. Constant in *E*_*c*,*intra*_, *C* ~ 0.1 could decrease down to ~0.01 (one-order) depending on the experiments.

We have to discuss here other important interactions among the 2D layers. These are the two kind Coulomb interactions, namely intra- (within one 2DEG layer) and inter- (between the 2DEG layers) ones. They are represented as *E*_*c*,*intra*_ ~ *C* (*e*^*2*^/*κ* *l*_*B*_) and *E*_*c*,*inter*_ ~ (*e*^*2*^/*κ* *d*) for intra- and inter-layer interactions, respectively, where *d* (~*t*_*QW*_) is a distance between the 2DEG layers and *C* the constant typically ≤0.1. *E*_*c*,*intra*_ is indeed an *E*_*g*_ itself discussed above and varies depending on *B* as shown in Fig. 6 when *C* ~ 0.1. Actually, *E*_*c*,*inter*_ decreases to the same order with *E*_*c*,*intra*_ only by assuming the relatively wide (*t*_QW_ > ~80 nm) well. In other words, in the narrow well with *t*_QW_ < ~80 nm, strong inter-layer interaction will destroy the fragile FQH states. This origin should be an important reason to explain the appearance of traces of FQHE shoulders/plateaus rather in the wider well (A, B, and C) samples.

Relevant important energy parameter is a *Δ*_*SAS*_, which is an energy gap between the symmetric-antisymmetric energy states in the wide well. This gap becomes large as the distance (*d*) between the bilayer 2DEGs decreases. It is difficult to determine this value in our heterojunction, since in the frequency analysis for the low field *R*_*xx*_, also the peak splitting due to the strong Rashba SOI^34^ can often appear and masks the *Δ*_*SAS*_ splitting. So that, we estimate *Δ*_*SAS*_ in our system simply by comparing with the results obtained in GaAs wide-single well quantum well (WSQW) samples^12,13^, where *ν* = 1/2 plateau has been observed for the first time. Taking well width and inter-layer electron tunneling into account, they have obtained *Δ*_*SAS*_ ~ 5 K for *d* ~ 50 nm. So that, in our samples A–C, *Δ*_*SAS*_ ~ 0.5 K since *d* ~ 100 nm, although the confinements at the both sides of the well are rather loose due to the small effective mass of the 2DEGs (this means the reduction of the distance *d* between the 2DEGs). We can thus conclude that the widths themselves seem sufficiently large to suppress the tunneling and hence *Δ*_*SAS*_ is small enough not to destroy the fractional nature in our samples A–C. This might be an another reason that the traces of fractional behavior have been found in rather wide well samples.

Finally, we make a short comment about the observation of the even denominator shoulders or plateaus in our samples. As is well known already from twenty years ago, even denominator FQH states, mainly *q*/*2* as well as odd denominator (*q*/*3*, *q*/*5* etc) ones have been widely reported also in bilayer system by many groups^12–14^. Although in this sense, the findings of *q*/*2* related phenomena in our samples are not curious. However, it is not clear at present that our samples are satisfying the more rigid condition^50^ suggested by a window in (*d*/*l*_*B*_) ~ 4 − 7 and *α* = *Δ*_*SAS*_/(*e*^*2*^/*ε* *l*_*B*_) ~ 0.05 − 0.1 space or not. These might be the problems discussed in the next article.

## Conclusion

We have studied QHE phenomena in 2DEG bilayer system formed in wide In_0.75_Ga_0.25_As quantum wells and found the fractional QH plateaus/shoulders under some specific conditions for the first time. They are likely sample dependent as if they are the “finger print” of the samples. We have prepared an imbalanced areal density pair in the bilayer 2DEGs as well as a controlled realization of bilayer-monolayer transition by a gate-voltage application. By combining these conditions, we can create a quantum limit for at least one of the 2DEG and hence a hysteretic process, in which *R*_*xx*_ and *R*_*xy*_ varies via the meta-stable states between the integer plateaus. This stochastic process is the origin of the “fingerprint” and seems the important reason that we were able to observe the FQHE-related signals even in alloy InGaAs systems at relatively high temperatures, despite a general negative perspective about sample disorder and FQHE activation energy criteria.

## Methods: High-Field Measurements

Low temperature and high-field MR measurements have been done by using the superconductive magnet in the usual refrigerator in the National Institute for Materials Science (NIMS) Japan and typical temperature and high-field conditions are ~1.6 K and ≤15 Tesla, respectively. Measurements are carried out in a standard low-frequency AC method with low-noise pre-amplifiers and lock-in amplifiers. Diagonal and Hall resistances, *R*_*xx*_ and *R*_*xy*_, are recorded as a function of magnetic field *B* with a parameter of V_g,top_.

## Supplementary information

Fractional quantum Hall effects in In0.75Ga0.25As bilayer electron systems observed as “Finger print”
