# Supplementary material for: Fractional quantum Hall effects in In0.75Ga0.25As bilayer electron systems observed as “Finger print”
Source: Sci Rep. 2019 May 15;9:7446. doi: 10.1038/s41598-019-43290-8 (PMC6520355; doi:10.1038/s41598-019-43290-8)
Supplement: Supplementary file 1 — Fractional quantum Hall effects in In0.75Ga0.25As bilayer electron systems observed as “Finger print” [file 41598_2019_43290_MOESM1_ESM.pdf]

Fractional quantum Hall effects in $In_{0.75}Ga_{0.25}As$ bilayer electron systems observed as "Finger print"

Syoji. Yamada*[,1] and Akira. Fujimoto[1]
[1]Osaka Institute of Technology
5-16-1, Omiya, Asahi-ku Osaka, 535-8585 Japan
Siro. Hidaka[2]
[2]LT Center, Osaka University
1-1, Machikaneyama, Toyonaka, Osaka 560-0043, Japan
Masashi. Akabori[3]
[3]Japan Advanced Institute of Science and Technology
1-1, Asahidai, Nomi, Ishikawa, 923-1292 Japan
, Yasutaka. Imanaka[4] and Kanji. Takehana[4]
[4]National Institute for Materials Science
3-13, Sakura, Tsukuba, Ibaraki, 305-0003 Japan

*shoji.yamada@oit.ac.jp

Supplementary Figure S1

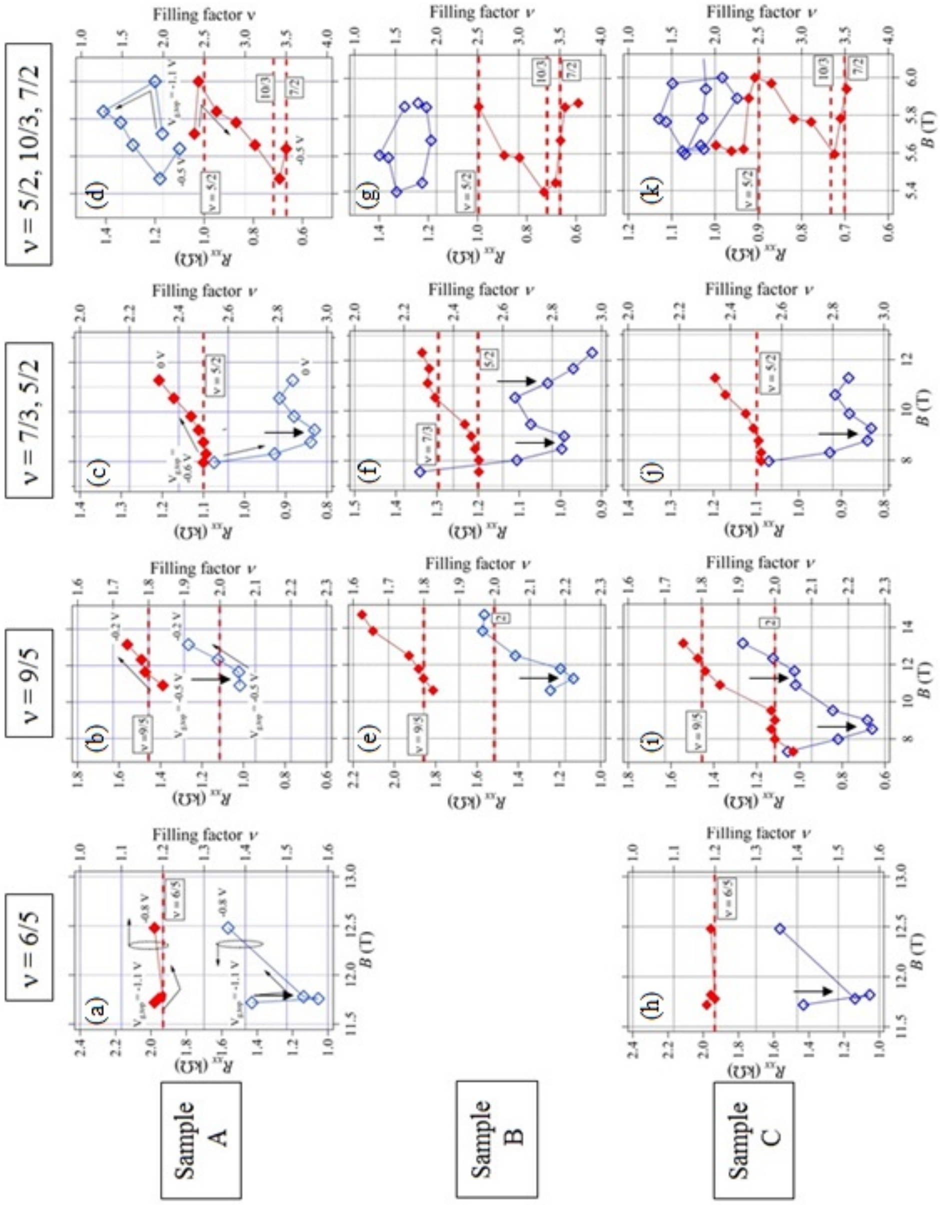

Figure S1 Three sets of R_{xx} dip – R_{xy} shoulder (plateau) pairs as functions of magnetic field (*B)* observed at various filling factors, ν ~ 6/5, ~9/5, ~7/3 + ~5/2, ~5/2 + ~10/3 corresponding to three different samples. The first set are the panels (a) – (d) in the top row and they are created from the data sets in the ellipsoids, (a) – (d) in Fig. 2(a) and (b). The second set including the panels (e) - (g) in the middle row corresponds to the MR data measured in sample B. The third set (panels (h) – (k)) in the bottom row corresponds to the data in sample C. Note the ν values approach to some fractional ones and also R_{xx}s decrease or show minima (arrows) at the same *B* regions.

Supplementary Figure S2

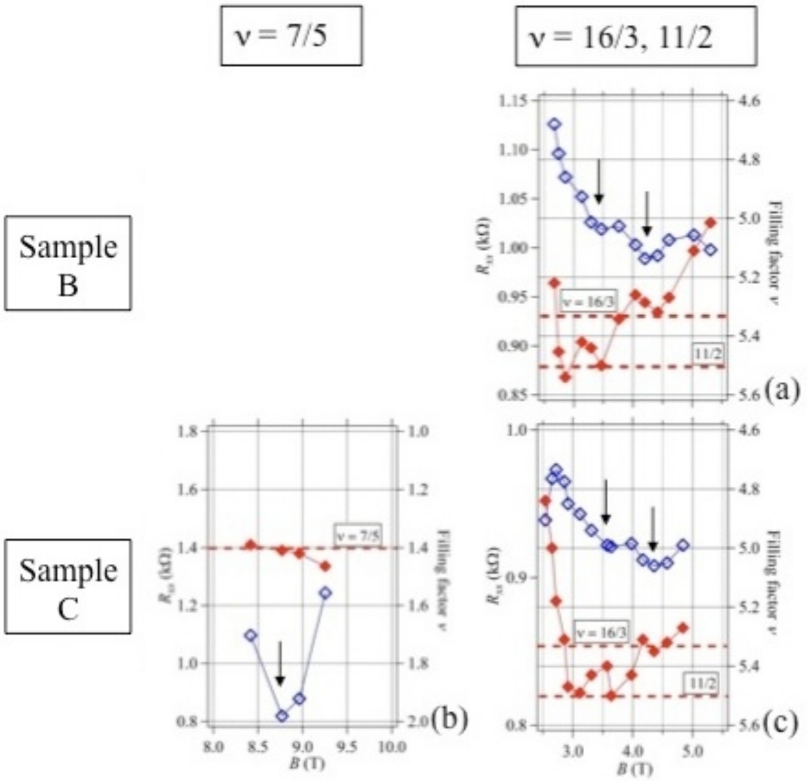

Figure S2 Two sets of R_{xx} dip – R_{xy} shoulder (plateau) pairs as functions of magnetic field (*B)* observed at filling factors, $\nu \sim 7/5$, ~16/3 and ~11/2 corresponding to two different samples. R_{xx} dip and R_{xy} shoulder pairs at n ~~16/3 and ~11/2 are similarly observed in between the samples B and C (in between thevpanels (a) and (c)).
